# Gingival lesion leading to a diagnosis of angiosarcoma

**DOI:** 10.1002/jgf2.397

**Published:** 2020-11-01

**Authors:** Yasuhiro Nakano, Yosuke Sazumi, Yuki Mizuta, Hiroyuki Sakae, Fumio Otsuka

**Affiliations:** ^1^ Department of General Medicine Okayama University Graduate School of Medicine, Dentistry and Pharmaceutical Sciences Okayama Japan

**Keywords:** echocardiography, gingiva, heart, malignant tumor, metastasis, sarcoma

## Abstract

A man who presented with a gingival tumor was finally diagnosed with angiosarcoma. It is important for primary care physicians to perform checkups of the oral cavity since oral lesions can lead to a diagnosis of serious systemic diseases.

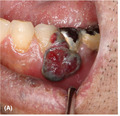

A 62‐year‐old man presented with a left lower gingival mass lasting for 2 months. The mass was prone to bleeding. He also had general malaise and dyspnea on exertion. He had a past history of myxofibrosarcoma of the right wrist that was surgically resected 15 years ago. A physical examination revealed a 10‐mm gingival mass in the left mandible (Figure 1A) with left cervical lymphadenopathy, breath sound attenuation and mild pitting edema in the bilateral legs. A chest radiography demonstrated mild cardiomegaly and bilateral pleural effusions. Echocardiography revealed moderate pericardial effusions, right ventricular collapse suggesting cardiac tamponade, and a mass in the right atrium (Figure 1B). Contrast‐enhanced computed tomography (CT) was performed due to suspicion of a malignant tumor, and nodular lesions in the left mandible, right atrium, lungs, esophagus, liver, and spleen were revealed. Positron emission tomography‐computed tomography (PET/CT) showed ^18^F‐fludeoxyglucose (FDG) uptake in the multiple bones in addition to the lesions on CT with a maximum standardized uptake value of 44.8 in the right atrial mass (Figure 1C,D). Although a primary cardiac tumor was considered because of the largest cardiac lesion, biopsy of the lesion was not performed due to its high invasiveness. Biopsies of the gingival and esophageal lesions revealed only reactive inflammation, but a liver biopsy finally revealed a pathological diagnosis of angiosarcoma. The patient received multidisciplinary therapy including chemotherapy and radiation for the cardiac lesion but died 3 months later.

**Figure 1:**
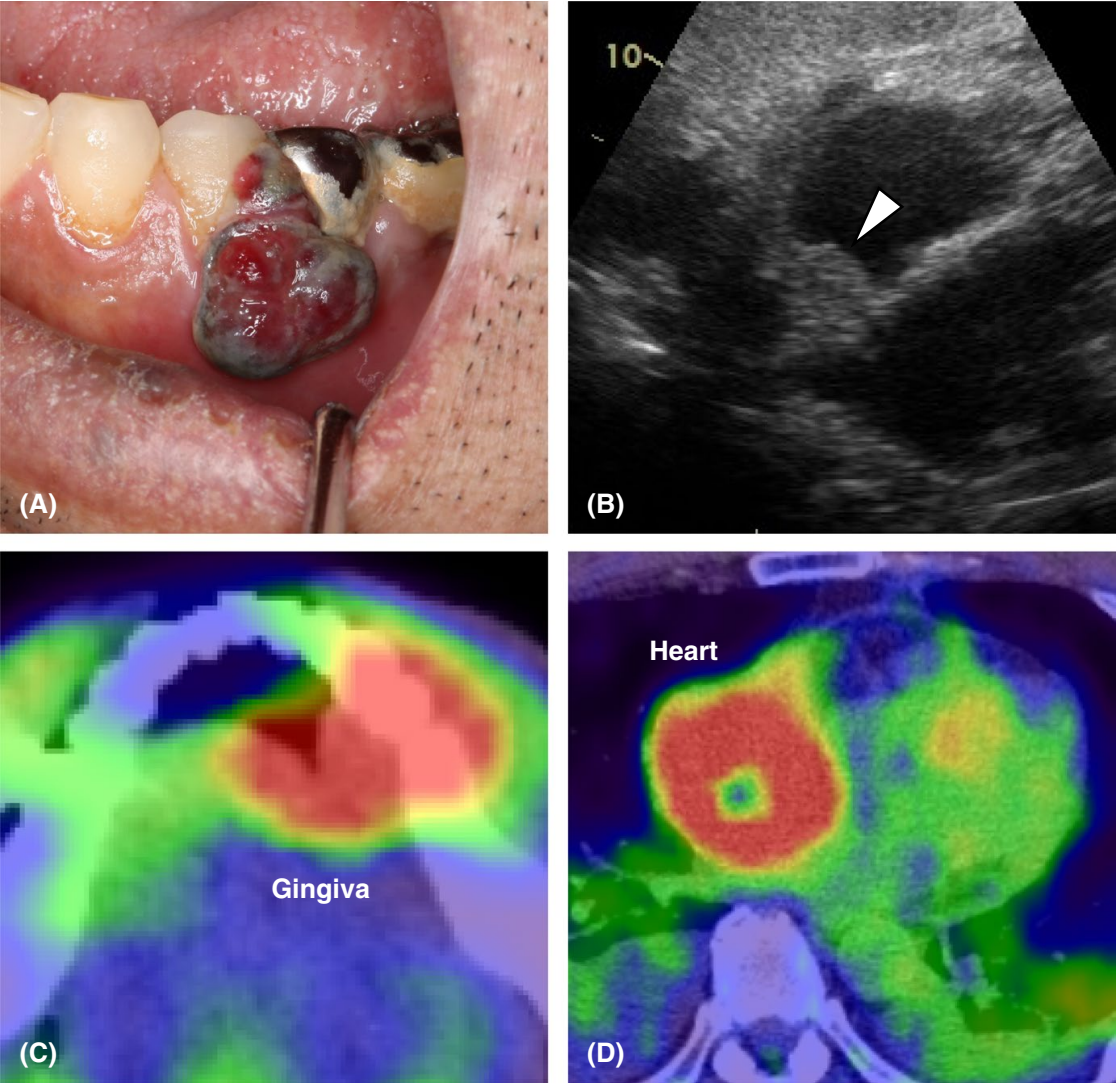
Appearance of the left lower gingival mass (A). Echocardiogram showing a mass in the right atrium (B). FDG‐PET/CT images showing a gingival to mandibular lesion on the left side (C) and a right atrial mass (D) with FDG uptake. FDG, ^18^F‐fludeoxyglucose; PET/CT, Positron emission tomography‐computed tomography.

Angiosarcoma is a highly aggressive soft tissue sarcoma. While it generally arises in the skin of the head and neck, approximately 5% of cases occur in the heart,^1^ the most common type of cardiac sarcoma. Cardiac angiosarcoma is commonly found in the right atrium and tends to infiltrate the myocardial wall and adjacent structures, resulting in pericardial effusion, cardiac tamponade, and ultimately cardiac rupture. Patients with cardiac angiosarcoma have an extremely poor prognosis with an average survival time of only 4 months. Early detection of cardiac sarcoma is difficult, but echocardiography is the most convenient and least invasive method for identifying cardiac tumors.^2^

Metastasis to the oral cavity occurs in only about 1% of oral neoplasms, the most frequent site of which is the mandible followed by the gingiva and tongue. Clinical presentations differ depending on the metastatic site. Patients complain of swelling, pain, and paresthesia in cases of mandible metastasis and a gingival mass and bleeding in cases of gingival metastasis. Oral metastasis occurs in cases of advanced neoplasms including lung, prostate, and breast cancers,^3^, ^4^ and metastatic angiosarcoma in the oral cavity is extremely rare. Oral metastases of angiosarcoma from the skin, breast, and lung have been reported, and the prognosis for such cases is worse than that for primary oral angiosarcoma.^5^ Although oral metastases have an overall poor prognosis, one fourth of them can be the initial sign of occult diseases.^4^ Since oral lesions can lead to a diagnosis of serious systemic diseases, it is important for primary care physicians to perform checkups of the oral cavity.

## CONFLICT OF INTEREST

The authors have stated explicitly that there are no conflicts of interest in connection with this article.
